# Stability and Species Specificity of Renal VEGF-A Splicing Patterns in Kidney Disease

**DOI:** 10.1371/journal.pone.0162166

**Published:** 2016-09-06

**Authors:** R. J. Turner, M. Eikmans, I. M. Bajema, J. A. Bruijn, H. J. Baelde

**Affiliations:** 1 Department of Pathology, Leiden University Medical Center, Leiden, the Netherlands; 2 Department of Immunohematology and Blood Transfusion, Leiden University Medical Center, Leiden, the Netherlands; Peking University First Hospital, CHINA

## Abstract

Vascular endothelial growth factor A (VEGF-A) is essential for maintaining the glomerular filtration barrier. Absolute renal levels of VEGF-A change in patients with diabetic nephropathy and inflammatory kidney diseases, but whether changes in the renal splicing patterns of VEGF-A play a role remains unclear. In this study, we investigated mRNA splicing patterns of pro-angiogenic isoforms of VEGF-A in glomeruli and whole kidney samples from human patients with kidney disease and from mouse models of kidney disease. Kidney biopsies were obtained from patients with acute rejection following kidney transplantation, patients with diabetic nephropathy, and control subjects. In addition, kidney samples were obtained from mice with lupus nephritis, mice with diabetes mellitus, and control mice. The relative expression of each VEGF-A splice variant was measured using RT-PCR followed by quantitative fragment analysis. The pattern of renal VEGF-A splice variants was unchanged in diabetic nephropathy and lupus nephritis and was stable throughout disease progression in acute transplant rejection and diabetic nephropathy; these results suggest renal VEGF-A splicing stability during kidney disease. The splicing patterns were species-specific; in the control human kidney samples, VEGF-A 121 was the dominant isoform, whereas VEGF-A 164 was the dominant isoform measured in the mouse kidney samples.

## Introduction

Vascular endothelial growth factor A (VEGF-A) is a pro-angiogenic glycoprotein in the platelet-derived growth factor family. VEGF-A is essential for the survival, proliferation, and differentiation of endothelial cells.[1, 2] In the kidney, VEGF-A is produced by visceral epithelial cells (podocytes) and transported across the glomerular basement membrane, ultimately reaching the glomerular endothelial cells, where VEGF-A plays a pivotal role in maintaining the glomerular filtration barrier by regulating the survival and structure of the endothelial cells.[3, 4] VEGF-A also regulates the structure and survival of podocytes via an autocrine loop.[5, 6] Many studies have highlighted the importance of VEGF-A under a variety of conditions; for example, in mice, both downregulation and upregulation of VEGF-A expression in developing glomeruli can result in severe glomerular abnormalities.[7] Moreover, overexpressing VEGF-A in the podocytes of adult mice causes proteinuria and structural changes in the glomeruli.[8] Conversely, inhibiting VEGF-A expression in adult mice causes severe thrombotic glomerular injury.[9] Together, these results provide compelling evidence that VEGF-A expression must be tightly regulated in order to maintain the integrity of the glomerular filtration barrier.

In patients with kidney disease, renal levels of VEGF-A change during disease progression, particularly in patients with diabetic nephropathy (DN), in which a reduction in renal VEGF-A production is correlated with disease progression.[10] Also, changes in renal VEGF-A levels are seen in inflammatory-based renal diseases; patients with lupus nephritis have increased plasma levels of VEGF-A, but renal VEGF-A mRNA levels are decreased and correlate with deterioration of renal function.[11, 12] During acute rejection of kidney allografts, the renal expression of VEGF-A and its receptors is increased.[13]

As these studies show, quantitative VEGF-A levels change in kidney diseases. However, qualitative changes in VEGF-A expression—specifically, changes in mRNA splicing patterns—can also play a role in pathophysiology, for example in the placenta in preeclampsia.[14] Changes in the splicing of anti-angiogenic splicing variants of VEGF-A, the VEGF-A_b_ variants, are involved in the development of kidney damage; however, whether changes in the pro-angiogenic splice variants of VEGF-A occur during kidney disease is currently unknown.[15]

The *VEGF-A* gene contains eight exons that are alternatively spliced, yielding at least seven distinct pro-angiogenic splice variants. The splice variants VEGF-A 121, 165, and 189 are the most abundantly expressed isoforms in humans. The same isoforms are expressed in mice, but the murine splice variants yield isoforms that are one amino acid shorter (i.e., VEGF-A 120, 164, and 188, respectively). The proteins encoded by these three splice variants differ with respect to their transport, storage, and signaling efficacy. For example, only the longer isoforms (i.e., VEGF-A 165 and 189) contain exons 6 and 7, which encode a heparin-binding site that enables VEGF-A to bind with the extracellular matrix.[16] These two isoforms—but not the VEGF-A 121 isoform—are also essential for embryonic development, as they inhibit epithelial cell apoptosis.[17] In addition, the VEGF-A 165 isoform includes the portion of exon 7 that encodes a binding site for neurophilin, a co-receptor for VEGF-A that enhances intracellular signaling.[18]

Based on the studies discussed above, the various pro-angiogenic isoforms of VEGF-A have distinct properties. Although total renal VEGF-A levels are altered in kidney disease, the role of each isoform is not currently known. To address this issue, we investigated qualitative changes in VEGF-A splicing patterns in a range of renal pathologies, including DN, acute renal transplant rejection, and lupus nephritis, with capillary electrophoresis. In addition, because the distribution of VEGF-A isoforms in the kidneys of healthy humans and rodents has not been examined with this method before, we also investigated VEGF-A splicing patterns in kidney samples obtained from healthy mice and humans.

## Materials and Methods

### Samples

VEGF-A splice variants were measured in kidney tissues obtained from 14 human donor kidneys.[10] Laser microdissection was used to isolate the glomeruli for assessing the glomeruli-specific splicing patterns of VEGF-A.

Kidney biopsies were collected from 28 patients with both diabetes type 2 and DN and used to measure the renal splicing pattern of VEGF-A. The clinical characteristics of this patient cohort have been described previously.[10] Laser microdissection was used to isolate the glomeruli. In addition, five kidney samples were obtained from mice with streptozotocin-induced diabetes (five weeks after induction), and five kidney samples were obtained from age-matched control mice; these samples were used to examine VEGF-A isoform expression in an early stage of DN.[19]

Kidney biopsies were obtained from 123 patients with acute rejection of a transplanted kidney;[20] these samples were used to investigate the renal splicing pattern of VEGF-A and were evaluated for acute and chronic lesions in accordance with the Banff classification system.[21]

Kidney samples were obtained from five healthy control mice and were used to examine the renal splicing pattern of VEGF-A. To assess the splicing patterns of VEGF-A in various organs, the lungs, lymph nodes, and spleens were obtained. Furthermore, kidney samples were obtained from a lupus nephritis mice model described and used to measure VEGF-A isoform expression at various time points. [22]

### Ethics

Human samples were obtained from archived patient material from biopsies performed for routine clinical procedures. None of the transplant donors were from a vulnerable population and all donors or next of kin provided informed consent that was freely given; patients visiting Dutch hospitals are actively informed of the ‘opt-out’ system regarding research with their archived material, this ‘Code of conduct for responsible use of residual material’ applies to all studies in the Netherlands using archived patient material. Only biopsies from patients who did not opt-out have been included in this study. Samples were collected and processed anonymously and according to the medical ethics committee of the Leiden University Medical Center and the ‘Code of conduct for responsible use of residual material’; approval from the institutional ethics board "Commissie Medische Ethiek LUMC" is given automatically to all studies following this code and correct adherence to the code is checked on a regular basis by national health care inspection.

Animal studies were approved by the DEC (Animal Experiments Committee) of the Leiden University Medical Center; the project code of this study is 13163. The care and use of all mice in this study was carried out in accordance with the Dutch law on animal testing. To ameliorate suffering, mice were anesthetized with isoflurane and sacrificed before collecting the kidneys.

### Primer design

We designed primers that amplify all VEGF-A isoforms. The human and mouse primer sets were designed to anneal to exon 3 and exon 8, which are present in all VEGF-A mRNA isoforms. When amplified using these primers, each isoform produces a unique-length PCR product. The primer sequences are given in S1 Table.

### PCR and capillary electrophoresis

RNA was isolated from the samples using Trizol (Life Technologies, San Francisco, CA). cDNA was synthesized using AMV reverse transcriptase (Roche, Basel, Switzerland). For PCR, FAM-labeled forward primers were used. The PCR products were subjected to capillary electrophoresis using an ABI 3100 fragment analyzer (Life Technologies). The ROX 500 dye was used as a size standard. The results were analyzed using GeneMapper, and the relative level of each VEGF-A isoform was calculated by dividing the peak height of the isoform of interest by the total sum of all isoform peaks. An example of a GeneMapper analysis is shown in S1 Fig.

### cDNA synthesis using specific primers

To test for a possible replication bias towards synthesizing cDNA from shorter isoform templates, we performed a one-step PCR using specific primers and mRNA isolated from glomerular samples obtained from three healthy humans. We also performed a serial dilution series of the resulting cDNAs.

### Western blot analysis

Lysates from prepared from two human control kidney samples and from a HEK293 cell line. Sample buffer containing 2-mercaptoethanol was added to the lysates, and the samples were loaded on a 12.5% polyacrylamide gel. Electrophoresis was performed at 100 V for 1.5 hours. The gel was transferred to a membrane following semi-dry blotting overnight. VEGF-A was visualized on the membrane using a rabbit polyclonal anti-VEGF antibody (SC-152; Santa Cruz Biotechnology, Dallas, TX) and the Odyssey red anti-rabbit antibody (Li-Cor Biotechnology, Lincoln, NE).

### Statistical analyses

Differences between groups were compared using either the unpaired *t*-test (for normally distributed data) or the Mann-Whitney *U* test (for data that were not distributed normally). Differences between >2 groups were analyzed using the one-way ANOVA. Differences with a *p*-value <0.05 were considered statistically significant. All analyses were performed using the SPSS statistics software package (version 20; IBM, Armonk, NY).

## Results

### VEGF-A splicing patterns in healthy human glomeruli and whole kidney samples

**Fig 1:**
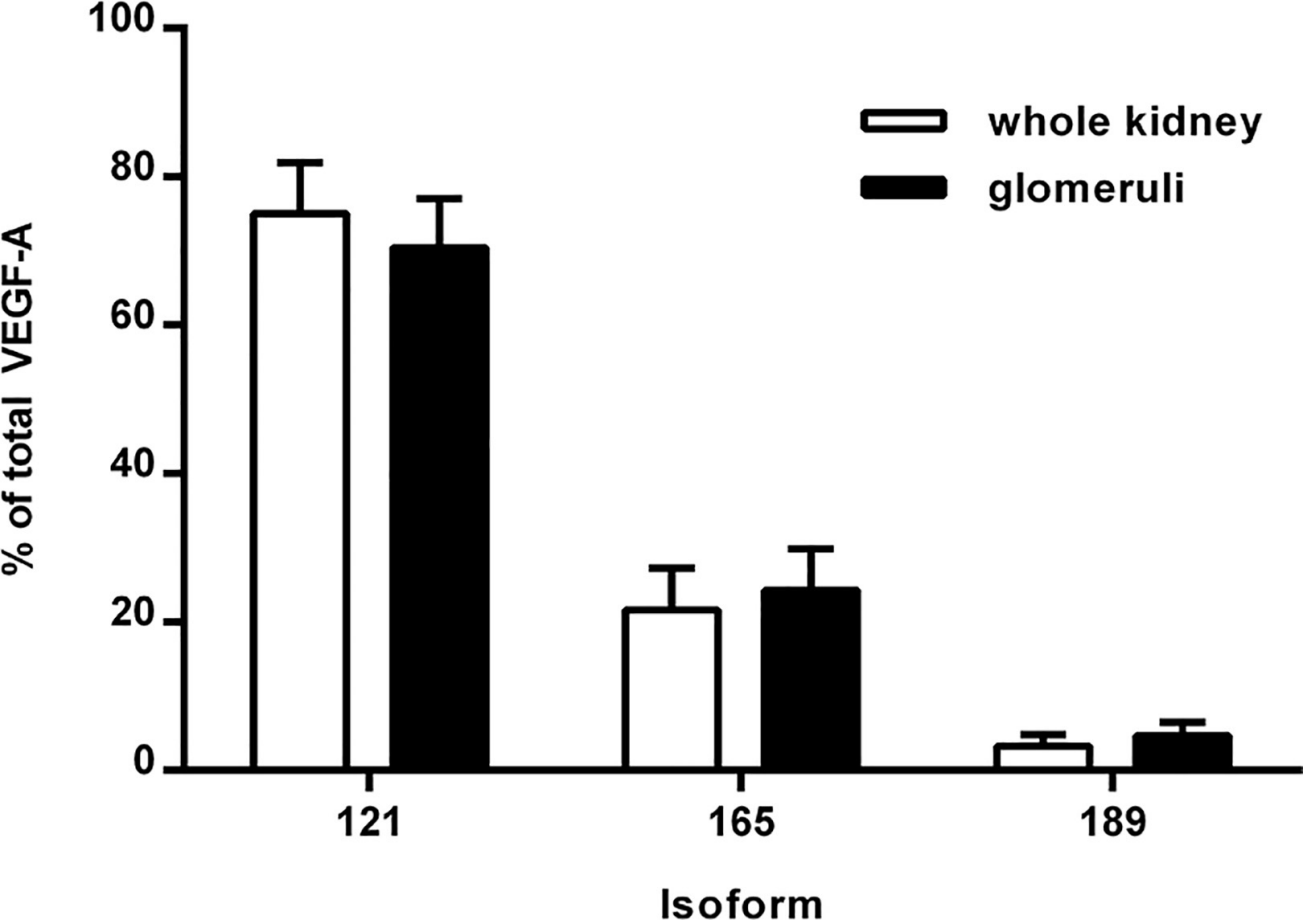
VEGF-A splicing patterns in healthy kidneys and glomeruli. The VEGF-A 121, 165, and 189 splice variants (isoforms) were measured in total kidney tissue and glomeruli (isolated using laser microdissection) obtained from healthy human subjects.

We found that VEGF-A 121, 165, and 189 isoforms were all expressed in the kidneys and glomeruli of healthy human controls, and VEGF-A 121 was the most abundant isoform (Fig 1). Specifically, in the glomeruli, VEGF-A 121, 165, and 189 comprised 70%, 24%, and 5%, respectively, of the total VEGF-A pool; in the whole kidney tissue samples, VEGF-A 121, 165, and 189 comprised 75%, 22%, and 3%, respectively (Fig 1). In some samples, the 181 isoform represented <1% of the total VEGF-A pool.

### VEGF-A splicing patterns in kidney disease

We next examined the VEGF-A splicing patterns in whole kidney tissue samples and microdissected glomeruli obtained from patients with moderate and advanced stages of DN. In these samples, we found a similar VEGF-A splicing pattern as in the healthy control samples. Specifically, VEGF-A 121, 165, and 189 comprised 78%, 20%, and 2%, respectively, of the VEGF-A pool in the whole kidney samples, and 72%, 22%, and 5%, respectively, of the VEGF-A pool in the glomeruli. As in the healthy samples, in a few of the disease samples, VEGF-A 181 represented <1% of the total VEGF-A pool. These patterns of VEGF-A splice variants were relatively stable, regardless of creatinine levels, proteinuria, or renal fibrosis.

Compared to healthy controls, the renal VEGF-A splicing patterns were slightly different in the renal transplant patients with acute rejection; in these patients, VEGF-A 121, 165, and 189 comprised 64%, 30%, and 5%, respectively, of the total VEGF-A pool (*p*<0.01 versus control for all three isoforms). In addition, the VEGF-A 145 and 181 isoforms were also detected in the patient samples, although both isoforms were present in extremely low levels (i.e., <1% of the total VEGF-A pool). The VEGF-A splicing patterns were stable in these patients, regardless of the Banff criteria or the degree of interstitial fibrosis.

**Fig 2:**
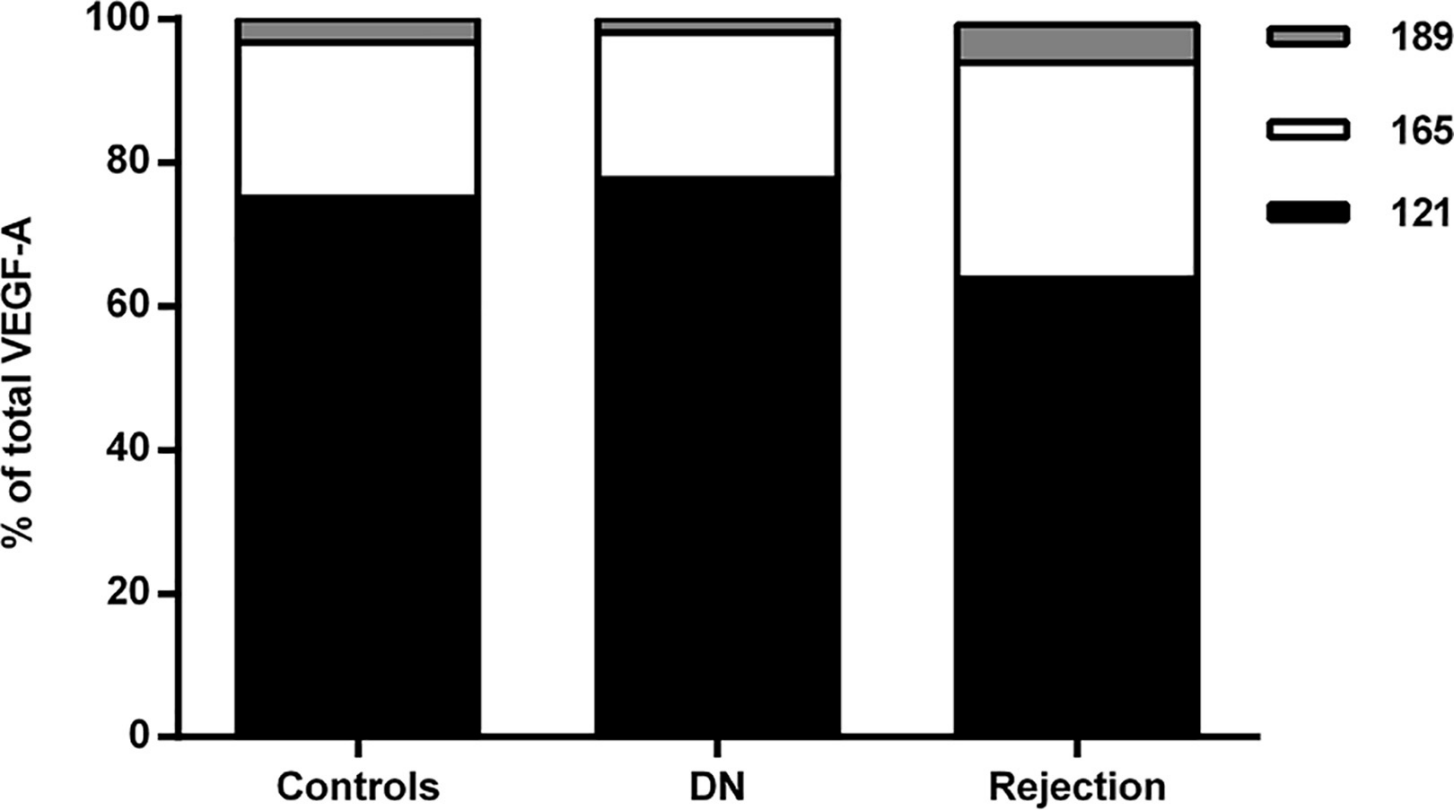
VEGF-A splicing patterns in kidney tissue obtained from healthy subjects, patients with DN, and patients with acute renal transplant rejection. The VEGF-A 121, 165, and 189 isoforms were measured in the indicated samples. DN, diabetic nephropathy.

The distribution of the three principal VEGF isoforms in the kidneys of healthy controls, patients with DN, and patients with acute rejection is summarized in Fig 2.

### VEGF-A isoform distribution in mouse kidney tissues

**Fig 3:**
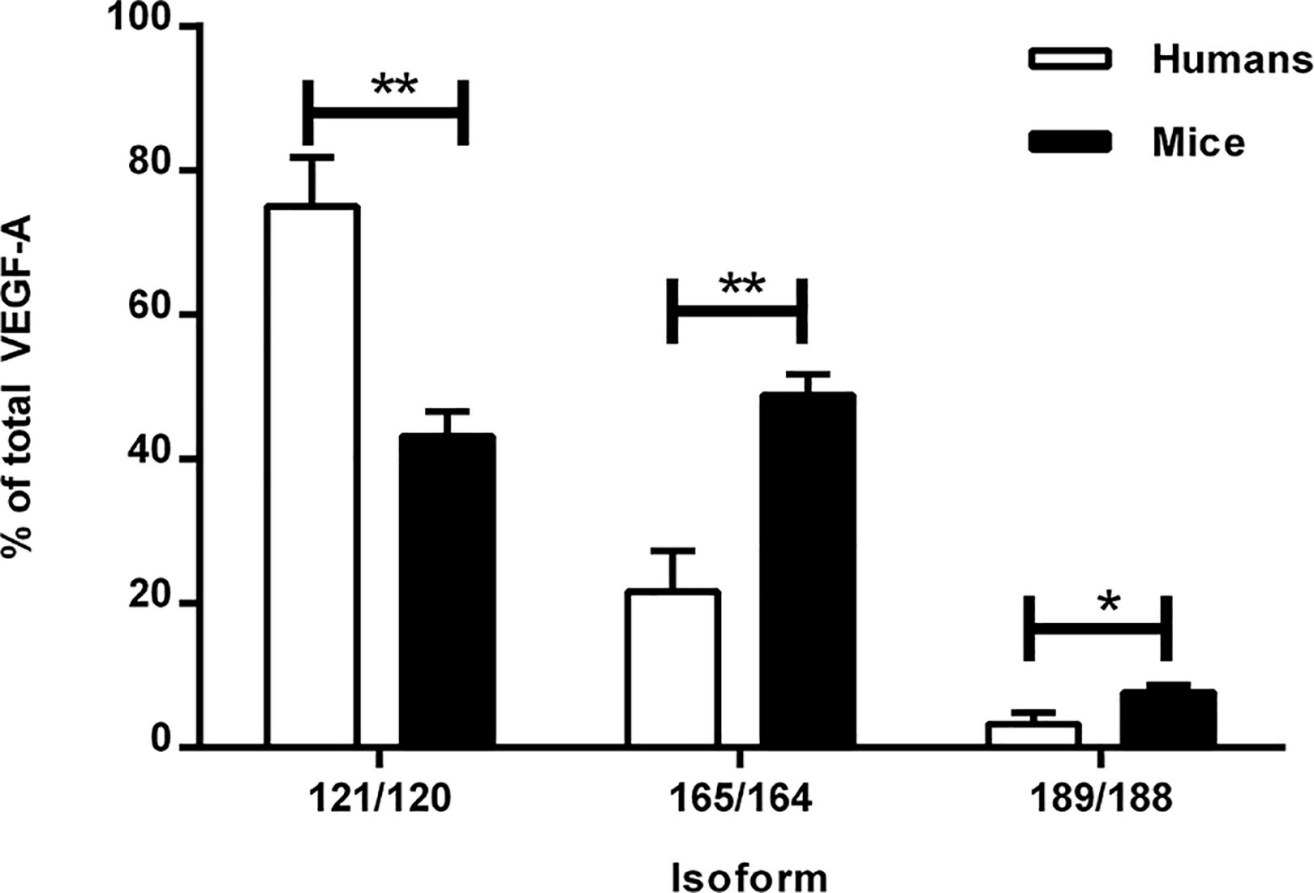
VEGF-A splicing patterns in kidney tissue obtained from healthy mice and humans. The VEGF-A 121, 165, and 189 isoforms were measured in kidney biopsies obtained from healthy human subjects, and the corresponding VEGF-A isoforms (VEGF-A 120, 164, and 188, respectively) were measured in kidney tissue and obtained from healthy mice. *p<0.001.

**Fig 4:**
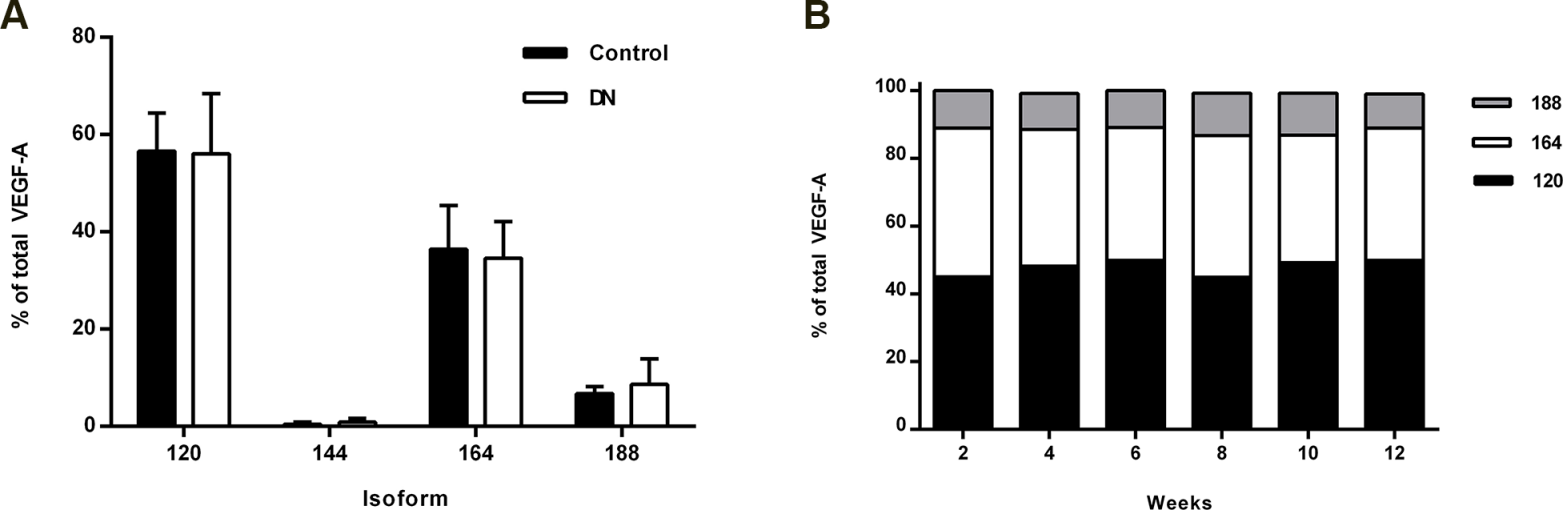
VEGF-A splicing patterns in whole kidney tissue obtained from mice with DN, healthy age-matched control mice and mice with lupus nephritis. **A** The VEGF-A 120, 144, 164, and 188 isoforms were measured in whole kidney tissue obtained from mice with early-stage DN and age-matched control mice. The two groups did not differ significantly with respect to any isoform measured (*p*>0.05). **B** Kidney samples were collected from mice at the indicated time points after lupus nephritis was induced. The glomeruli were isolated, and the VEGF-A 120, 164, and 188 isoforms were measured. The distribution of VEGF-A isoforms was unchanged over the duration of the experiment.

Next, we measured the VEGF-A splicing patterns in mouse kidney samples. Interestingly, we found that the renal splicing pattern in mice was significantly different than the pattern seen in human samples. In mice, VEGF-A 164 was the most abundant isoform, comprising approximately 48% of the total VEGF-A pool (*p*<0.001 versus human). The relative proportions of VEGF-A 120, 144, and 188 were 44% (*p*<0.001), <1%, and 7% (*p*<0.001), respectively (Fig 3). In kidney samples obtained from mice with diabetes, the VEGF-A splicing pattern was similar to healthy, age-matched control mice (Fig 4). The VEGF-A splicing pattern was similar in mice with lupus nephritis, and the pattern did not change over time (Fig 4).

### cDNA synthesis using specific primers

Similar splicing patterns were obtained using specific primers in a one-step PCR compared to the experiments with random primers, and serial dilution of the cDNA had no effect on the VEGF-A splicing pattern (S2 Fig).

### Distribution of VEGF-A isoforms in various mouse organs

We found different VEGF-A splicing patterns in tissue samples obtained from different organs from the same mouse (S3 Fig). For example, in lung tissue, VEGF-A 120, 164, and 188 comprised 53%, 28%, and 18%, respectively, of the total VEGF-A pool. In the thymus of the same mouse, these isoforms comprised 58%, 29%, and 13%, respectively, of the total VEGF-A pool.

### Western blot analysis

Next, to confirm expression of the VEGF 121, 165, and 189 isoforms, we performed a western blot analysis using kidney samples obtained from two healthy human controls (S4 Fig). Both samples contained three bands that corresponded with the predicted molecular weight of the three VEGF-A isoforms. In addition, we performed a western blot analysis on HEK293 cell lysate and found bands corresponding to the VEGF-A 121 and 165 isoforms; VEGF 189 was not detected in the cell lysate (S4 Fig).

## Discussion

We demonstrate stability of the renal VEGF-A splicing pattern in renal pathology; specifically, splicing patterns were stable regardless of several histopathological parameters of disease progression in human patients with DN and acute renal transplant rejection, as well as in a mouse model of lupus nephritis. We also found that the pattern of VEGF-A splicing in the kidney is species-specific; notably, the VEGF-A 121 splice variant is the most abundant isoform in the human kidney, whereas the VEGF-A 164 splice variant is the most abundant isoform in mice.

In our study, we focused on the splicing pattern of VEGF-A in kidney disease. Changes in the splicing patterns of other alternatively spliced genes play a role in the development of kidney disease. For example, changes in the splicing pattern of fibronectin can play a role in the pathogenesis of glomerulosclerosis and tubulointerstitial fibrosis by modulating the immune response and scar formation.[23] Further, Oltean et al. recently discovered that changes in splicing of anti-angiogenic VEGF-A isoforms are involved in the progression of diabetic nephropathy. Because the renal splicing pattern of pro-angiogenic VEGF-A remains stable irrespective of disease progression of DN or during acute transplant rejection, it is unlikely that a disruption in the distribution of pro-angiogenic VEGF-A isoforms plays a major role in the development of kidney disease.

On the other hand, changes in the distribution of these isoforms in the kidney could serve a protective role with respect to renal pathology. The kidney’s inability to modify the distribution of VEGF-A isoforms to a pattern that protects endothelial cells under inflammatory conditions could facilitate the progression of kidney disease. For example, an upregulation of the VEGF-A 165 and VEGF-A 189 isoforms confers a robust, prolonged protective effect in glomerular endothelial cells.[18] However, because the pattern of VEGF-A isoforms does not appear to change in kidney disease, VEGF-A 121—which has relatively weak intracellular signaling strength—remains the predominant isoform, regardless of the underlying pathology.[18] Therefore, an increase in total VEGF-A production might not provide the sufficient conditions to rescue glomerular endothelial cells during renal pathology.

In our study, we quantified the pattern of VEGF-A splicing at the mRNA level. However, it is possible that the pathological changes in DN are not related to changes in VEGF-A mRNA expression and/or splicing, but may be related to post-translational modifications of VEGF-A. For example, the levels of plasmin-activator inhibitor are increased in patients with diabetes type 2; thus, plasmin-mediated post-translational cleavage of the VEGF-A 189 isoform could be dysregulated in the kidneys of patients with DN.[24, 25] This dysregulation would lead to a decrease in the cleavage products of VEGF-A 189, which may play a role during disease phases with decreased angiogenic potential.[18]

Our findings in healthy human kidney tissue are comparable to previous findings by Whittle et al. and Simon et al., who detected VEGF-A 121 and 165 as the main isoforms expressed in kidney tissue with PCR and gel electrophoresis.[26, 27] Our findings contradict the findings of a previous study by Bortoloso et al., in which a correlation was observed between albumin excretion, histological changes in the glomerulus, and the levels of VEGF-A 121 and 165 levels in patients with DN.[28] This discrepancy might be due to the differences in methods used between the two studies; for example, Bortoloso et al. used PAGE followed by silver staining to quantify the various VEGF isoforms, whereas we used capillary electrophoresis. However, two lines of evidence suggest that our approach is both sensitive and robust. First, we synthesized cDNA using specific primers in order to exclude any bias for replicating the shorter isoforms. We found no difference in cDNA that was synthesized using specific primers or random primers. Second, we found distinct patterns of VEGF-A isoforms in different organs obtained from one mouse, demonstrating that our approach is sufficiently sensitive to detect differences in the VEGF-A splicing pattern. Based on these results, we conclude that regulation of VEGF-A splicing is tissue-specific, but is not altered under the pathological conditions investigated in our study.

In mouse kidney, VEGF-A 164 is the predominant VEGF-A isoform. Given the difference in splicing patterns between humans and mice, these species might differ with respect to the regulation of VEGF-A expression in the kidney. Therefore, the pathogenesis of VEGF-A‒related diseases may differ between patients and mouse models. Mouse models that overexpress the VEGF-A 164 isoform have often been used to investigate kidney diseases[8, 29, 30]; however, because VEGF-A 121 is the predominant renal VEGF-A isoform in humans, these models may not fully represent the clinical situation in patients.

The results of our study may create opportunities to study the transport of VEGF-A in the kidney. In the kidney, VEGF-A is produced by podocytes, and it must cross the glomerular basement membrane in order to reach the glomerular endothelial cells. The glomerular basement membrane is comprised primarily of heparan sulfate proteoglycans, to which the longer VEGF-A isoforms (i.e., 165 and 189) can bind.[18, 31] Therefore, these heparan sulfate proteoglycans may play a role in the storage of VEGF-A in the kidney and in the transport of VEGF-A across the glomerular basement membrane. However, because VEGF-A 121 does not bind to heparan sulfate proteoglycans, it is freely diffusible.[18] Therefore, the transport of VEGF-A across the glomerular basement membrane and the storage of VEGF-A in the human kidney may be regulated by other mechanisms.

## Supporting Information

S1 Fig Example data from of a GeneMapper analysis of capillary electrophoresis. The GeneMapper analysis output of a capillary electrophoresis experiment is shown. The three blue peaks labeled 121, 165, and 189 indicate the VEGF-A 121, 165, and 189 isoforms, respectively.
(TIF)

S2 Fig Measuring VEGF-A isoforms in glomeruli with a one-step PCR with specific primers and serial dilutions. The VEGF-A 121, 165, and 189 splice variants (isoforms) were measured with a one-step PCR with specific primers and subsequent serial dilutions in glomeruli (isolated using laser microdissection) obtained from three healthy human subjects.
(TIF)

S3 Fig VEGF-A splicing patterns in several organs in one mouse. The VEGF-A 120, 144, 164, and 188 isoforms were measured in the indicated organs from a single healthy mouse.
(TIF)

S4 Fig Western blot analysis of human VEGF-A isoforms in healthy kidney tissue. The VEGF-A 121, 165, and 189 isoforms were detected using an anti-VEGF antibody that recognizes all three isoforms. Two control human kidney samples (A and B), as well as HEK293 cells (H), were lysed and subjected to western blot analysis. Note that all three isoforms were detected in the two kidney sample lysates, whereas only two isoforms (VEGF-A 121 and 165) were detected in the HEK293 cell lysate. The numbers at the left indicate the size marker (M; in kDa). The arrows and numbers at the right indicate the expected sizes of the indicated VEGF-A isoforms.
(TIF)

S1 File VEGF isoform expression in and clinical characteristics of cases and controls. (XLSX)

S1 Table Sequences of the primers used to amplify the human and mouse VEGF mRNA splice variants. (DOCX)
