# Supplementary figures and images for: Stability and Species Specificity of Renal VEGF-A Splicing Patterns in Kidney Disease
Source: PLoS One. 2016 Sep 6;11(9):e0162166. doi: 10.1371/journal.pone.0162166 (PMC5012578; doi:10.1371/journal.pone.0162166)

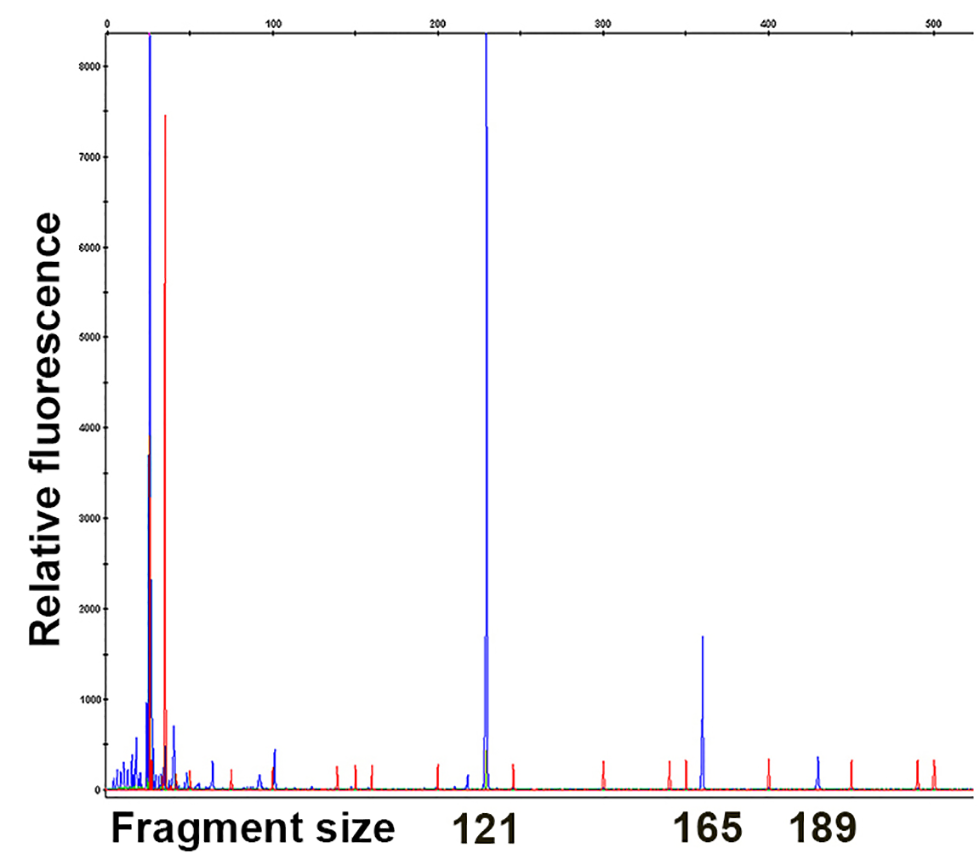

Supplement: S1 Fig — The GeneMapper analysis output of a capillary electrophoresis experiment is shown. The three blue peaks labeled 121, 165, and 189 indicate the VEGF-A 121, 165, and 189 isoforms, respectively. (TIF) [file pone.0162166.s001.tif]

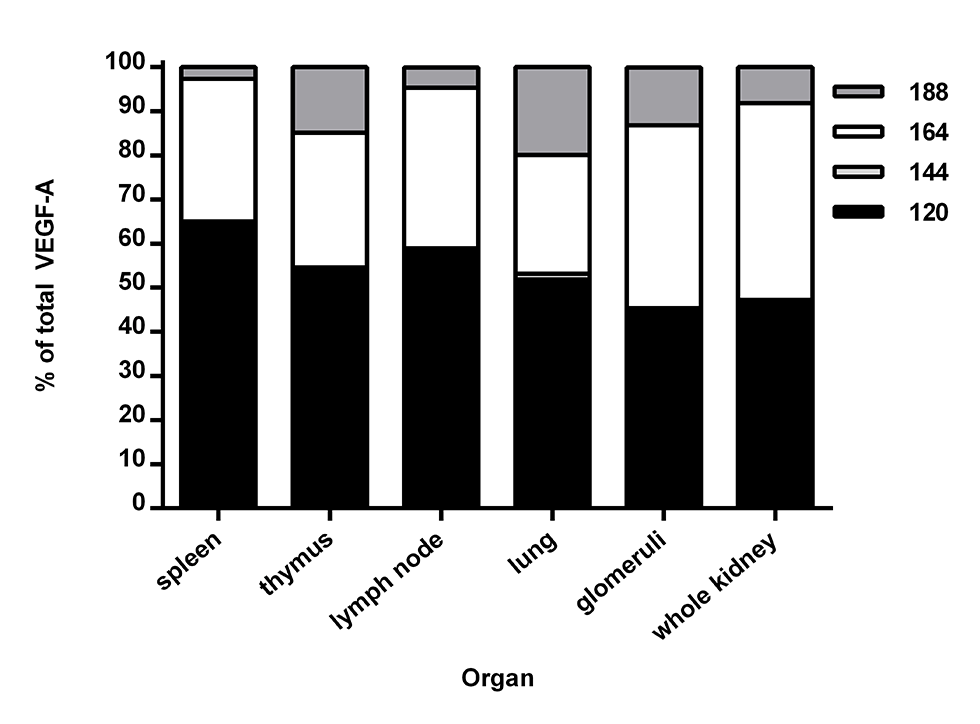

Supplement: S3 Fig — The VEGF-A 120, 144, 164, and 188 isoforms were measured in the indicated organs from a single healthy mouse. (TIF) [file pone.0162166.s003.tif]

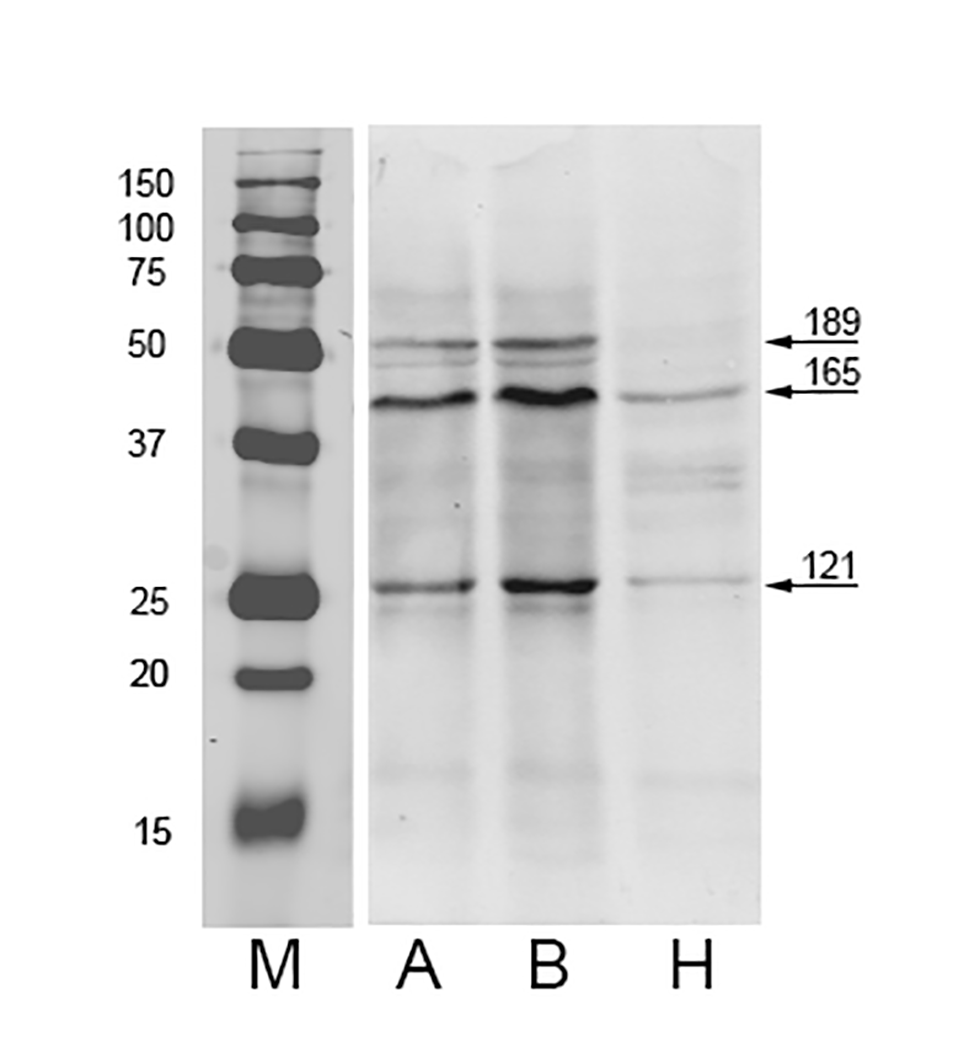

Supplement: S4 Fig — The VEGF-A 121, 165, and 189 isoforms were detected using an anti-VEGF antibody that recognizes all three isoforms. Two control human kidney samples (A and B), as well as HEK293 cells (H), were lysed and subjected to western blot analysis. Note that all three isoforms were detected in the two kidney sample lysates, whereas only two isoforms (VEGF-A 121 and 165) were detected in the HEK293 cell lysate. The numbers at the left indicate the size marker (M; in kDa). The arrows and numbers at the right indicate the expected sizes of the indicated VEGF-A isoforms. (TIF) [file pone.0162166.s004.tif]
