# Supplementary material for: Stability and Species Specificity of Renal VEGF-A Splicing Patterns in Kidney Disease
Source: PLoS One. 2016 Sep 6;11(9):e0162166. doi: 10.1371/journal.pone.0162166 (PMC5012578; doi:10.1371/journal.pone.0162166)
Supplement: S1 Table — (DOCX) [file pone.0162166.s006.docx]

| **Species** | **Primer** | **Sequence** |
| --- | --- | --- |
| **Human** | Forward | 5'FAM-TGTGCCCCTGATGCGATGCG-3' |
|  | Reverse | 5'-TCCTTCCTCCTGCCCGGCTC -3' |
| **Mouse** | Forward | 5'-CTGTGTGCCGCTGATGCGCT-3' |
|  | Reverse | 5'FAM-TCGCCCTCCGGACCCAAAGT-3' |

**S1 Table. Sequences of the primers used to amplify the human and mouse VEGF mRNA splice variants.**
